# Clinical characteristics and prognosis of bacteraemia during postoperative intra-abdominal infections

**DOI:** 10.1186/s13054-018-2099-5

**Published:** 2018-07-07

**Authors:** Adel Alqarni, Elie Kantor, Nathalie Grall, Sebastien Tanaka, Nathalie Zappella, Mathieu Godement, Lara Ribeiro-Parenti, Alexy Tran-Dinh, Philippe Montravers

**Affiliations:** 1 Département d’Anesthésie-Réanimation, CHU Bichat-Claude Bernard, APHP, Paris, France; 2 King Saud University, Riyadh, Saudi Arabia; 3 Université Paris Diderot, Press Sorbonne Cité, Paris, France; 4 Laboratoire de Microbiologie, CHU Bichat-Claude Bernard, APHP, Paris, France; 5 INSERM UMR 1137, Paris, France; 6 INSERM UMR 1188, Saint-Denis de la Réunion, France; 7 Service de Chirurgie Générale, CHU Bichat-Claude Bernard, APHP, Paris, France; 8 INSERM UMR 1148, Paris, France; 9 INSERM UMR 1152, Paris, France

**Keywords:** Intra-abdominal infection, Postoperative infection, Bacteraemia, Outcome

## Abstract

**Background:**

Bloodstream infections of abdominal origin are usually associated with poor prognosis. We assessed the clinical and microbiological characteristics of critically ill patients admitted to the intensive care unit (ICU) for postoperative intra-abdominal infection (PIAI) and analysed the influence of bacteraemia on their outcome.

**Methods:**

All consecutive PIAI patients admitted to the ICU between 1999 and 2014 were prospectively analysed. Bacteraemic patients (at least one positive blood culture in the 24 h preceding/following surgery) were compared with non-bacteraemic patients. Demographic characteristics, underlying disease, severity scores at the time of reoperation, microbiological results, therapeutic management, outcome, and survival were recorded. Results are expressed as median (interquartile range (IQR)) or proportions.

**Results:**

Overall, 343 patients (54% male, 62 (49–73) years old) with PIAI were analysed, including 64 (19%) bacteraemic patients. Immunosuppression and cancer were more frequent in bacteraemic patients (*p* < 0.001 in both cases). No difference between groups was observed for the characteristics of initial surgery. Time to reoperation, site, and cause of PIAI were similar in both groups. At the time of reoperation, Sequential Organ Failure Assessment (SOFA) score was higher in bacteraemic patients (8 (6–10) versus 7 (4–10); *p* < 0.05). A predominance of Gram-positive (34%) and Gram-negative (47%) bacteria were recovered from blood cultures (polymicrobial bacteraemia in 9 (14%) patients and bacteraemia involving multidrug-resistant organisms in 14 (22%) patients). In multivariate analysis, risk factors for bacteraemia were immunosuppression or cancer, high SOFA score, and *E. coli* in peritoneal samples. Bacteraemia did not impact the management (with similar results for the adequacy of antibiotic therapy, anti-infective agents used, de-escalation or duration of therapy in both groups). Neither hospital mortality nor morbidity criteria differed between groups. Risk factors for mortality in multivariate analysis were urgent initial surgery, high Simplified Acute Physiology Score (SAPS) II score and documented antifungal therapy, but not perioperative bacteraemia.

**Conclusions:**

In this ICU population, bacteraemia did not change the overall management of patients with PIAI. Our data suggest that bacteraemic patients do not require a specific management.

## Background

Bacteraemia is a major cause of morbidity and mortality in critically ill patients [1–3], defined as primary when no source of infection is identified or secondary to dissemination of pathogens from other sites of infection [4]. One of the most common causes of secondary bacteraemia is intra-abdominal infection, accounting for 11–15% of all secondary cases in critically ill patients [4, 5].

Bacteraemia secondary to intra-abdominal surgical site infection is not uncommon, with a reported incidence of 10–26% at the time of reoperation [6, 7] and increased rates in the case of delayed diagnosis [8], septic shock [6], or multidrug-resistance [9]. Patients who develop postoperative intra-abdominal infection (PIAI) are at higher risk of adverse outcome and mortality. Interestingly, no specific data are available in the literature concerning bacteraemia during PIAI. In a retrospective single-centre cohort study of 96 critically ill patients, De Waele et al. found that the mortality of patients with bacteraemia of abdominal origin was high (62.5%) and higher than the Acute Physiology and Chronic Health Evaluation (APACHE) II-based expected mortality rate [10]. These investigators found that older age and renal failure were independent risk factors of death. However, only 39% of their population had secondary peritonitis and the proportion of PIAI cases was not specified.

Another controversial issue concerns the duration of antibiotic therapy in bacteraemic patients. Prolonged therapy has been reported in patients treated for bacteraemia [11], which can be explained by the fear of increased treatment failure rates in the case of short-course antibiotic therapy [12]. However, insufficient evidence is available in patients with peritonitis.

The objective of this study was to analyse the impact of bacteraemia on morbidity and mortality in critically ill PIAI patients. We hypothesised that outcome is determined by both clinical and microbiological factors in this population.

## Methods

### Study population

Between January 1999 and December 2014, all consecutive intensive care unit (ICU) patients admitted to our surgical ICU for management of a first episode of PIAI were prospectively included in a database. This observational study was conducted according to the terms and regulations of the local institutional review boards (CEER CHU Bichat, Paris Diderot University, Paris, agreement 10–008). According to French Law, no informed consent was required since this observational study did not modify the physician’s treatment decisions.

### Data collection

Bacteraemic patients were defined as having at least one positive blood culture in the 24 h preceding or following surgery for PIAI. In case of positive blood culture for coagulase-negative staphylococci (CNS), two positive blood cultures with the same microorganism and the same antibiotic susceptibility were required to establish the diagnosis of bacteraemia or one positive blood culture matching the same microorganism with the same antibiotic susceptibility in the peritoneal samples. Medical charts were retrospectively reviewed. Demographic characteristics, underlying medical conditions, and their severity according to the McCabe score [13] were recorded. The type of initial surgery and presence of antimicrobial therapy prior to PIAI surgery were also recorded. Anatomical location above or below the transverse mesocolon and source of PIAI were noted.

Severity scores (Simplified Acute Physiology Score (SAPS) II and Sequential Organ Failure Assessment (SOFA) scores) were obtained at the time of reoperation for PIAI [14, 15]. Temperature, white blood cell counts, serum creatinine, and SOFA score were assessed on the day of reoperation (day 0) and at days 3 and 7 after surgery for patients still in the ICU. Differences (delta) between the SOFA scores observed on day 3 and day 0 were calculated. Patients meeting the following three criteria on day 3 were arbitrarily defined as improving: SOFA score ≤ 3 points, white blood cell counts ≥ 5000 and ≤ 15,000 cells/mm^3^, and temperature ≥ 36 °C and ≤ 38 °C. Similar analyses were performed at day 7 to compare changes in these criteria between day 7 and day 0.

### Microbiological examinations

Blood cultures were systematically collected during the management of PIAI. All blood culture bottles were handled using the laboratory’s blood culture system (BD BACTEC™; Becton-Dickinson). Positive blood cultures drawn within 24 h before and after reoperation for PIAI were noted. Diagnostic procedures on all positives bottles included Gram stain and bacterial isolation and identification using standard bacteriology techniques. The antibiotic susceptibility of each strain was determined using the disk diffusion method (http://www.sfm-microbiologie.org).

Peritoneal fluid samples were systematically collected during surgery and were immediately sent to the bacteriology laboratory. Samples were processed according to the laboratory’s standard methods. Plates were incubated for 48 h at 35 °C. All morphologically distinct colonies were identified and tested for antibacterial susceptibility by the disk diffusion method (http://www.sfm-microbiologie.org) [7]. Multidrug-resistant (MDR) bacteria were defined as those resistant to three or more antimicrobial classes [16].

### Anti-infective therapy

Empirical antibiotic therapy (EAT) was started at the time of reoperation for PIAI. The choice of therapy took into account the severity of the case, previous antibiotic therapies, and local epidemiology. According to French guidelines, EAT included a combination of broad-spectrum beta-lactams such as piperacillin/tazobactam or imipenem/cilastatin associated with aminoglycosides and vancomycin [17]. EAT, changes at the time of documented therapy including escalation and de-escalation, and total duration of therapy were recorded [7]. Empirical antifungal agents were administered when a high risk of fungal infection was suspected and adapted to the identification results of peritoneal samples [17, 18].

### Outcome

The primary outcome was ICU mortality from any cause. Medical and surgical complications, suspicion of persistent sepsis [19, 20], additional reoperations for persistence of the initial infection or superinfections (including MDR bacteria), and death between the day following surgery and discharge from hospital were assessed. Duration of ICU stay and mechanical ventilation were also assessed.

### Statistical analysis

Results are expressed as median and interquartile range (IQR) or number and proportions. Statistical analysis was performed with SAS© 9.4 (SAS Institute, Cary, NC, USA). Comparisons between bacteraemic and non-bacteraemic patients used Chi-square test and Fisher’s exact test for discrete variables and unpaired Wilcoxon tests for quantitative variables. Risk factors for bacteraemia and death were assessed by univariate analysis and unadjusted odds ratio (OR) and 95% confidence intervals (CIs) were calculated. Variables with *p* values less than 0.15 in univariate analysis were introduced as dependent factors into two multivariate logistic regression analyses using a stepwise selection method, and the collinearity between predictors was analysed. Bacteraemia constituted the outcome of the first model, and ICU mortality constituted the outcome of the second model. Bacteraemia was forced in the model of ICU mortality as our primary factor of interest until the end of the selection process. In addition, the year of inclusion was forced in both analyses to take the time effect into account. Logistic models were evaluated for discrimination with the C-statistic and for calibration with the Hosmer-Lemeshow test.

## Results

### Patient characteristics

Overall, 343 PIAI patients including 64 (19%) bacteraemic patients were analysed. The demographic characteristics and underlying diseases at the time of diagnosis were similar in both groups, except for increased proportions of immunosuppression and cancer in bacteraemic patients (Table 1). Surgical characteristics were similar in both groups, while bacteraemic patients were slightly more severe at the time of diagnosis as illustrated by the SOFA score (Table 1). The median SAPS II score slowly increased from 46.0 in 1999 to 54.0 in 2014.

**Table 1:** Characteristics and clinical findings of patients with positive blood cultures and patients without bacteraemia

Variables	All patients (*n* = 343)	Bacteraemic patients (*n* = 64)	Patients without bacteraemia (*n* = 279)	Odds ratio	95% CI	*p* value
Demographic characteristics and underlying diseases						
Age (years), median (IQR)	62 (49–73)	63 (52–74)	61 (48–73)	0.92	(0.53–1.58)	0.56
Male gender, *n* (%)	184 (54)	32 (50)	152 (54)	0.83	(0.49–1.44)	0.52
Fatal underlying disease, *n* (%)	110 (32)	29 (45)	81 (29)	2.02	(1.16–3.53)	0.012
Immunosuppression, *n* (%)	121 (35)	37 (59)	84 (30)	3.25	(1.85–5.71)	< 0.001
Diabetes mellitus, *n* (%)	59 (17)	12 (19)	47 (17)	1.14	(0.56–2.29)	0.72
Cancer, *n* (%)	126 (37)	35 (55)	91 (33)	2.49	(1.43–4.33)	< 0.001
Initial surgery						
Upper gastrointestinal tract surgery, *n* (%)	150 (44)	26 (41)	124 (44)	0.85	(0.49–1.48)	0.57
Septic or contaminated surgery, *n* (%)	123 (36)	25 (42)	98 (37)	1.20	(0.68–2.12)	0.53
Emergency procedure, *n* (%)	135 (39)	22 (34)	113 (41)	0.77	(0.43–1.35)	0.37
Antibiotics before reoperation for PIAI, *n* (%)	215 (63)	43 (67)	172 (62)	1.25	(0.70–2.22)	0.45
Interval between initial surgery and reoperation (days), median (IQR)	7 (4–12)	8 (4–15)	7 (4–11)	1.09	(0.63–1.89)	0.25
Severity criteria at the time of reoperation						
SAPS II score, median (IQR)	47 (35–59)	50 (38–63)	47 (34–58)	0.89	(0.52–1.54)	0.076
SOFA score, median (IQR)	8 (4–10)	8 (6–10)	7 (4–10)	1.10	(0.59–1.75)	0.034
Haemodynamic failure^a^, *n* (%)	224 (65)	46 (73)	178 (64)	1.53	(0.83–2.81)	0.16
Respiratory failure^a^, *n* (%)	154 (45)	30 (48)	124 (44)	1.13	(0.65–1.96)	0.65
Renal failure^a^, *n* (%)	83 (24)	16 (25)	67 (24)	1.05	(0.56–1.98)	0.86
Source of postoperative peritonitis and surgical observations						
Generalised infection, *n* (%)	79 (23)	20 (31)	59 (21)	1.69	(0.92–3.09)	0.08
Gastroduodenal source of infection, *n* (%)	74 (22)	11 (17)	63 (23)	0.71	(0.35–1.44)	0.34
Small bowel source of infection	88 (26)	13 (20)	75 (27)	0.69	(0.35–1.34)	0.27
Colonic or rectal source of infection, *n* (%)	97 (28)	20 (31)	77 (28)	1.19	(0.66–2.15)	0.56
Anastomotic leak, *n* (%)	118 (34)	25 (40)	93 (33)	1.31	(0.74–2.29)	0.34
Bowel perforation, *n* (%)	119 (35)	16 (25)	103 (37)	0.57	(0.30–1.05)	0.07
Abscesses, *n* (%)	61 (18)	15 (23)	46 (16)	1.55	(0.80–2.99)	0.19
No demonstrated cause, *n* (%)	49 (14)	11 (17)	38 (14)	1.31	(0.63–2.74)	0.46

^a^According to levels 3 or 4 of the SOFA score

*CI* confidence interval, *IQR* interquartile range, *SAPS* Simplified Acute Physiology Score, *SOFA* Sequential Organ Failure Assessment

### Microbiological results

Overall, 102 positive blood cultures were collected in bacteraemic patients, yielding 64 microorganisms with a median of 1 (range 1–8) positive blood cultures per patient (Table 2): 21 (33%) patients with aerobic Gram-positive bacteraemia, 30 (47%) patients with aerobic Gram-negative bacteraemia, 10 (16%) patients with anaerobic bacteraemia, and 6 (9%) patients with fungaemia. Polymicrobial bacteraemia was observed in 9 (14%) cases (including 2 aerobic Gram-negative organisms (*n* = 2), 2 aerobic Gram-positive organisms (*n* = 1), 2 anaerobes (*n* = 2), and 2 fungi (*n* = 1)). Among these organisms, 14 bacteraemic patients had MDR bacteria: 6 Gram-positive bacteria (3 methicillin-resistant CNS, 1 methicillin-resistant *S. aureus*, and 2 *E. faecium*) and 8 Gram-negative bacteria (6 ESBL or AmpC-producing *Enterobacteriaceae*, and 2 *Pseudomonas* spp). Among the microorganisms isolated from blood culture, 29 organisms in 27 patients were not isolated from surgical operative samples: *E. coli n* = 4, *Enterobacter* spp. *n* = 1, *Pseudomonas* spp. *n* = 3, enterococci *n* = 1, streptococci *n* = 4, CNS *n* = 1, *S. aureus n* = 3, and anaerobes *n* = 10.

**Table 2:** Microbiological results of peritoneal samples and antimicrobial therapy in bacteraemic and non-bacteraemic patients

Microbiological results	Bacteraemic patients (*n* = 64)		Patients without bacteraemia (*n* = 279)
	Blood cultures	Surgical samples	Surgical samples
Total number of microorganisms	75	215	807
Gram-negative aerobic bacteria, *n* (%)	32 (43)	97 (45)	312 (39)
*Enterobacteriaceae, n* (%)	27 (36)	86 (40)	254 (31)
*Escherichia coli*, *n* (%)	13 (17)	42 (20)	124 (15)
*Klebsiella spp.*, *n* (%)	3 (4)	10 (5)	34 (4)
*Enterobacter spp.*, *n* (%)	8 (11)	17 (8)	44 (5)
Other *Enterobacteriaceae*, *n* (%)	3 (4)	17 (8)	52 (6)
Non-fermenting Gram-negative bacteria, *n* (%)	4 ()	10 (5)	44 (5)
*Pseudomonas aeruginosa*, *n* (%)	3 (4)	9 (4)	39 (5)
Miscellaneous Gram-negative bacilli, *n* (%)	1 (1)	1 (0.5)	14 (2)
Gram-positive aerobic bacteria, *n* (%)	22 (29)	73 (34)	308 (38)
*Staphylococcus aureus*, *n* (%)	5 (7)	4 (2)	26 (3)
Coagulase-negative staphylococci, *n* (%)	5 (7)	8 (4)	50 (6)
*Streptococcus* spp., *n* (%)	8 (11)	21 (10)	75 (9)
*Enterococcus* spp., *n* (%)	3 (4)	38 (18)	151 (19)
Miscellaneous Gram-positive cocci, *n* (%)	1 (1)	2 (1)	6 (1)
Anaerobic bacteria, *n* (%)	14 (19)	22 (10)	76 (9)
*Bacteroides* spp., *n* (%)	12 (16)	18 (8)	46 (6)
Other anaerobes, *n* (%)	2 (3)	4 (2)	30 (4)
Multidrug-resistant bacteria, *n* (%)	14 (19)	26 (12)	113 (14)
Fungi, *n* (%)	7 (9)	23 (11)	110 (14)
*Candida albicans*, *n* (%)	3 (4)	13 (6)	71 (9)
Non-albicans Candida spp., *n* (%)	3 (4)	9 (4)	32 (4)

Proportions calculated as number of isolates

A total of 1022 microorganisms were isolated from peritoneal surgical samples (Table 2). Polymicrobial peritoneal cultures were reported in 58/64 (91%) bacteraemic and 228/279 (82%) non-bacteraemic patients, respectively (*p* = 0.09). The frequency of bacteraemia was increased in patients with *Enterobacteriaceae*, *E. coli*, and *E. faecalis* isolated from peritoneal samples (OR 2.25, 95% CI 1.12–4.52, *p* = 0.022; OR 2.12, 95% CI 1.22–3.70], *p* = 0.007; and OR 1.98, 95% CI 1.12–3.52, *p* = 0.019; respectively). The proportions of MDR bacteria cultured from peritoneal samples were similar between bacteraemic and non-bacteraemic patients (26 (41%) versus 113 (41%) patients, respectively; *p* = 0.50).

### Risk factors for bacteraemia

The risk factors for bacteraemia identified in univariate analysis are presented in Table 3. In multivariate analysis, after adjustment for the year of inclusion, we identified three risk factors independently associated with an increased risk of bacteraemia: immunosuppression or cancer, SOFA score at the time of reoperation, and *E. coli* isolated from surgical peritoneal samples (Table 3).

**Table 3:** Risk factors for bacteraemia in univariate and multivariate analysis

	Univariate analysis			Multivariable analysis		
Variables	Odds ratio	95% CI	*p* value	Adjusted odds ratio	95% CI	*p* value
Demographic characteristics and underlying diseases						
Fatal underlying disease	2.02	(1.16–3.53)	0.012			
Cancer or immunosuppression	2.64	(1.51–4.62)	< 0.001	2.59	(1.46–4.59)	0.001
Severity criteria at the time of reoperation						
SOFA score^a^	1.10	(0.59–1.75)	0.034	1.19^a^	(1.02–1.39)	0.002
Source of postoperative peritonitis and surgical observations						
Generalised peritonitis	1.69	(0.92–3.09)	0.08			
Interval between initial surgery and reoperation^a^	1.02	0.98–1.05	0.247			
Microbiological results of peritoneal surgical samples						
*Enterobacteriaceae*^b^	2.52	(1.23–5.20)	0.010			
*Escherichia coli*^b^	2.13	(1.22–3.71)	0.008	2.10	(1.19–3.73)	0.011

The model was adjusted on year of admission

Multivariate logistic regression with stepwise selection: the C-index of the model was 0.69 95% CI 0.61–0.77 and the Hosmer-Lemeshow test *p* value was 0.377

*CI* confidence interval, SOFA Sequential Organ Failure Assessment

^a^Per 1 day

^b^Per 2 points

### Anti-infective therapy

Empirical therapy did not differ between groups (Table 4). Similar proportions of adequacy of EAT were achieved in both groups. At the time of documented therapy, proportions of escalation, de-escalation, and combination therapy for the targeted therapy were not different between groups. In addition, the median duration of therapy was not different between groups (Table 4).

**Table 4:** Empirical and documented therapies in bacteraemic and non-bacteraemic patients

Anti-infective regimens	Bacteraemic patients (*n* = 64)	Patients without bacteraemia (*n* = 279)	*p* value
Empirical therapy			
Combination of EAT, n (%)	49 (77)	226 (81)	0.42
Imipenem/cilastatin, *n* (%)	22 (34)	69 (25)	0.12
Piperacillin tazobactam, *n* (%)	36 (56)	173 (62)	0.39
Vancomycin, *n* (%)	30 (47)	122 (44)	0.65
Aminoglycosides, *n* (%)	31 (48)	140 (50)	0.80
Antifungals, *n* (%)	21 (33)	101 (36)	0.61
Fluconazole, *n* (%)	17 (27)	89 (32)	0.45
Caspofungin, *n* (%)	3 (5)	8 (3)	0.43
Adequacy of EAT, *n* (%)	43 (67)	190 (68)	0.89
Documented therapy			
Antibiotic treatment de-escalation, *n* (%)	37 (58)	163 (58)	0.93
Antibiotic treatment escalation, *n* (%)	15 (23)	72 (26)	0.69
Combination of EAT, *n* (%)	38 (59)	190 (68)	0.19
Imipenem/cilastatin, *n* (%)	23 (36)	56 (20)	0.007
Piperacillin tazobactam, *n* (%)	20 (31)	98 (35)	0.55
Vancomycin, *n* (%)	16 (25)	90 (32)	0.25
Aminoglycosides, *n* (%)	12 (19)	36 (13)	0.22
Antifungals, *n* (%)	20 (31)	117 (42)	0.11
Fluconazole, *n* (%)	16 (25)	104 (37)	0.06
Caspofungin, *n* (%)	2 (3)	7 (3)	0.78
Duration of antibiotic therapy in days^a^, median (IQR)	10 (8–14)	10 (8–14)	0.89

*EAT* Empirical antibiotic therapy, *IQR* interquartile range

^a^Variable measured in surviving patients

In the group of bacteraemic patients, empirical therapy did not adequately target all bloodstream microorganisms in 11 (17%) cases. The organisms most frequently not adequately treated were methicillin-resistant CNS (*n* = 5), penicillinase-producing *Enterobacteriaceae* (*n* = 2), *Candida* spp. (*n* = 2), and *E. faecium* (*n* = 1). Interestingly, 8 of these patients also had inadequate therapy for peritoneal pathogens.

### Patient outcome and risk factors of death

Comparison of the clinical characteristics of bacteraemic patients and those without positive blood cultures did not reveal any significant difference on day 3 and day 7 (Table 5). A trend toward increased ICU mortality rate was observed in bacteraemic patients (*p* = 0.08).

**Table 5:** Clinical outcome in bacteraemic and non-bacteraemic patients

Clinical outcomes	Bacteraemic patients (*n* = 64)	Patients without bacteraemia (*n* = 279)	Odds ratio	95% CI	*p* value
On day 3 after surgery for PIAI					
Temperature ≥ 36 °C and ≤ 38 °C, *n* (%)	25/55 (45.4)	165/243 (67.9)	0.39	(0.21–0.71)	< 0.01
WBC count ≥ 5000 and ≤ 15,000/mm^3^, *n* (%)	20/55 (57.1)	108/243 (44.4)	0.71	(0.39–1.31)	0.27
SOFA score ≤ 3 points, *n* (%)	22/55 (40)	104/243 (42.8)	0.89	(0.49–1.62)	0.70
All three criteria, *n* (%)	7/55 (12.7)	42/243 (17.3)	0.69	(0.29–1.64)	0.54
Delta SOFA between day 3 and day 0, median (IQR)	1 (0–4)	2 (0–4)	1.00^a^	(0.91–1.11)	0.82
On day 7 after surgery for PIAI					
Temperature ≥ 36 °C and ≤ 38 °C, *n* (%)	29/44 (65.9)	140/200 (70)	0.82	(0.41–1.65)	0.59
WBC count ≥ 5000 and ≤ 15,000/mm^3^, *n* (%)	23/44 (52.3)	84/200 (42)	1.51	(0.78–2.91)	0.21
SOFA score ≤ 3 points, *n* (%)	22/44 (50)	112/200 (56)	0.78	(0.41–1.51)	0.46
All three criteria, *n* (%)	12/44 (27.3)	44/200 (22)	1.33	(0.63–2.79)	0.45
Delta SOFA between day 7 and day 0, median (IQR)	3.5 (1.2–6)	4 (1–6)	0.98^a^	(0.89–1.11)	0.93
Suspected persistent sepsis, *n* (%)	36 (58)	144 (53)	1.21	(0.69–2.11)	0.50
Reoperation, *n* (%)	29 (45)	122 (44)	1.02	(0.59–1.76)	0.81
Total number of reoperations, median (IQR)	1 (1–2)	2 (1–2)	0.88	(0.71–1.10)	0.21
Interval between surgery for PIAI and reoperation, median (IQR)	5 (2.5–7)	5 (2–8)	0.97	(0.87–1.08)	0.42
Main reasons for reoperation					
Septic shock/multiple organ failure, *n* (%)	11/29 (38)	47/122 (39)	0.97	(0.42–2.24)	0.95
Sepsis, *n* (%)	18/29 (62)	75/122 (62)	1.02	(0.44–2.36)	0.95
Haemorrhage, *n* (%)	2/29 (7)	9/122 (7)	0.93	(0.18–4.55)	1
Suspicion of bowel ischaemia, *n* (%)	2/29 (7)	26/122 (21)	0.27	(0.06–1.22)	0.11
Main intraoperative diagnoses at reoperation					
Anastomotic leak, *n* (%)	8/29 (28)	29/122 (24)	1.22	(0.48–3.04)	0.64
Bowel perforation, *n* (%)	4/29 (14)	25/122 (20)	0.62	(0.19–1.94)	0.60
Ischaemia, *n* (%)	2/29 (7)	23/122 (19)	0.31	(0.07–1.43)	0.17
Abscess or collections, *n* (%)	5/29 (17)	14/122 (11)	1.60	(0.53–4.89)	0.37
No identified cause, *n* (%)	5/29 (17)	10/122 (8)	2.33	(0.73–7.44)	0.16
Medical complications, *n* (%)	5 (8)	37 (14)	0.55	(0.20–1.47)	0.29
Duration of mechanical ventilation, median (IQR), days^b^	8 (4–15)	7 (3–15)	1.45	(0.71–2.95)	0.75
ICU stay, median (IQR), days^b^	12 (8–23)	15 (8–25)	0.96	(0.48–1.94)	0.39
Death in ICU, *n* (%)	26 (41)	82 (29)	1.64	(0.94–2.88)	0.08
Death in hospital, *n* (%)	26 (41)	88 (32)	1.48	(0.85–2.59)	0.16

*CI* confidence interval, *IQR* interquartile range, *PIAI* postoperative intra-abdominal infection, *SOFA* Sequential Organ Failure Assessment, *WBC* white blood cell

^a^Per 1 point

^b^Variable measured in survivors

The mortality rate was similar in bacteraemic patients with MDR bacteraemia and those without MDR bacteraemia (5/15 (33%) versus 22/49 (45%); *p* = 0.55). Moreover, similar proportions of medical and surgical complications were observed in both groups. Mortality was observed in 3/6 (50%) cases of fungaemia versus 23/58 (40%) of the other bacteraemic patients (*p* = 0.68). No significant difference in terms of outcome was observed between the 11 bacteraemic patients who did not receive adequate EAT against bloodstream isolates versus the adequately treated cases: persistent intra-abdominal sepsis in 7 (64%) versus 31 (58%), *p* = 1; one or more reoperations in 4 (36%) versus 26 (49%), *p* = 0.52; and death in the ICU in 6 (55%) versus 21 (40%), *p* = 0.50.

The risk factors for mortality identified in univariate analysis are presented in Table 6. In multivariate analysis, after adjustment for the year of inclusion and the presence of bacteraemia, the following factors were independently associated with a higher mortality rate: urgent initial surgery (*p* = 0.0003), SAPS II > 47 at the time of reoperation (*p* < 0.0001), and empirical antifungal therapy (*p* = 0.002), while de-escalation of documented therapy (*p* = 0.003) had a protective role.

**Table 6:** Univariate and multivariate analyses of risk factors of intensive care unit (ICU) death in the study population

	Univariate analysis			Multivariate analysis		
Parameters	Odds ratio	95% CI	*p* value	Odds ratio	95% CI	*p* value
Demographic characteristics and underlying diseases						
Age > 62 years	2.45	(1.55–3.90)	< 0.001			
McCabe Fatal	1.55	(0.96–2.49)	0.08			
Immunosuppression or cancer	1.83	(1.15–2.92)	0.01			
Urgent initial surgery	2.04	(1.29–3.23)	0.002	2.71	(1.53–4.81)	0.0006
Severity criteria at the time of reoperation						
Small bowel perforation	1.57	(0.95–2.59)	0.07			
SAPS II > 47 at reoperation	4.26	(2.61–6.94)	< 0.001	4.78	(2.71–8.45)	< 0.0001
Blood and peritoneal microbiological results						
Bacteraemia	1.48	(0.84–2.59)	0.16	1.62	(0.81–3.24)	0.169
Peritoneal samples involving						
Enterococci	1.71	(1.08–2.69)	0.019			
Non-fermenting Gram-negative Bacteria	2.01	(1.13–3.65)	0.019			
Streptococci	0.54	(0.30–0.96)	0.037			
Empirical and documented therapies						
Empirical imipenem therapy	1.66	(1.01–2.72)	0.044			
Empirical vancomycin therapy	1.56	(0.99–2.46)	0.050			
Empirical antifungal therapy	1.72	(1.08–2.74)	0.019	2.91	(1.62–5.22)	0.0003
De-escalation documented therapy	0.54	(0.34–0.85)	0.007	0.50	(0.28–0.89)	0.018
Documented therapy using piperacillin/tazobactam	1.74	(1.09–2.78)	0.018			
Documented antifungal therapy	1.67	(1.05–2.63)	0.026			

The model was adjusted on year of admission

Multivariate logistic regression with stepwise selection: the C-index for the model was 0.81 95% CI 0.76–0.86, the Hosmer-Lemeshow test *p* value was 0.02

*CI* confidence interval, *SAPS* Simplified Acute Physiology Score

## Discussion

In this single-centre cohort study of patients with PIAI, the incidence of perioperative bacteraemia was 19%. A trend toward increased mortality rate in bacteraemic patients was observed compared with non-bacteraemic patients, with no major changes in morbidity criteria. The factors associated with bacteraemia were the presence of immunosuppression, ongoing cancer, and high SOFA score. The microorganisms isolated from the surgical site did not differ between bacteraemic and non-bacteraemic patients except for an increased proportion of *E. coli* in bacteraemic patients. Adequate empirical antimicrobial therapy rates and duration of antibiotic therapy did not differ between bacteraemic and non-bacteraemic patients. High severity scores, initial emergency surgery, and antifungal therapy were independent risk factors for mortality in multivariate analysis.

Few data are available assessing the clinical consequences of secondary bloodstream infections in peritonitis. Most of the published reports do not distinguish community-acquired infections from healthcare-associated infections. During bacteraemia in surgical ICU patients, the factors associated with poor prognosis are the severity of acute illness at the onset of bacteraemia, the presence of organ dysfunction, and Gram-negative bacteria or *Candida* spp. infections [21]. One-third of cases of undiagnosed abdominal septic focus presented breakthrough infection or reappearance of positive blood cultures during antibiotic therapy [22]. Polymicrobial bacteraemia is usually suggestive of intra-abdominal sepsis, but other sites are also associated with several polymicrobial samples [10, 11]. Blood cultures yielding Gram-negative bacilli, enterococci, and anaerobes are indicative of gastrointestinal flora, but staphylococcal bacteraemia is also reported [6–9].

In our cohort, the identified criteria for recognising bacteraemic patients during PIAI were poorly discriminative and did not allow any specific management. As expected, the most severe cases were those at risk of bacteraemia. This point has been previously reported, as bacteraemia associated with intra-abdominal infections appears to be more frequent in patients with septic shock [6]. The size of the inoculum could also be of importance, as suggested by the increased risk of bacteraemia in the case of generalised peritonitis and peritoneal samples yielding *E. coli* cultures. Severe underlying disease and immunosuppression have been previously described as possible risk factors for bacteraemia, but these criteria were not assessed in surgical postoperative infections.

In our PIAI patients, bacteraemia was associated with limited clinical consequences. Clinical recovery, analysed on day 3 and day 7 after surgery, did not differ between groups. Our data confirm the observations of Havey et al., who reported early improvement of clinical parameters in patients treated for bacteraemia [11]. Our observations suggest an incidental nature of bacteraemia rather than a major threat. This assumption is also confirmed by the absence of any specific morbidity criteria in the postoperative course of bacteraemic patients compared with non-bacteraemic patients. Additional observations, such as the high mortality rates reported in the case of fungaemia and bacteraemia not targeted by empirical therapy, could be considered to be signals of interest. However, the number of cases was too small to draw any strong conclusions.

A poorer prognosis was expected in the cohort of bacteraemic patients. The 41% mortality rate in our study confirms the high mortality rate previously reported by De Waele et al. [10]. However, only a trend towards an increased mortality rate was observed in our bacteraemic patients compared with non-bacteraemic patients. This point was confirmed in univariate analysis, while other well-known factors, such as emergency surgery and initial severity, were identified as risk factors for mortality. Interestingly, the prognosis did not differ in the presence of fungaemia compared with bacteraemia. The small sample size of our cohort clearly affected the validity of these results. Surprisingly, the role of fungaemia in peritonitis cases has been rarely analysed in the literature, while fungaemia is a known factor of poor prognosis in many instances [23, 24]. Nevertheless, fungal infection might play a role in the prognosis, as indirectly reflected by documented antifungal therapy which was a strong criterion of poor prognosis in our cohort.

The need to treat all isolated microorganisms in these high-risk ICU patients is a subject of debate. Recently published guidelines have failed to address this issue [17, 25, 26]. The usual rule is to target all pathogens isolated from both blood and surgical samples. However, the pathogenic role of certain microorganisms such as staphylococci is debated. Some data suggest a pathogenic role of *S. aureus* [27]. On the contrary, the role of CNS as a contaminant on blood or surgical samples remains much more controversial. In order to limit the risk of contamination, we used strict selection rules for CNS bacteraemia and we assume that the selected isolates represent authentic pathogens. By means of these criteria, CNS represented only a limited proportion of the organisms isolated from bacteraemic patients. Among the 11 bacteraemic patients in whom empirical anti-infective therapy did not target all bloodstream microorganisms, almost half of the cases involved CNS. Interestingly, the outcome of these cases was not completely favourable, suggesting that discarding these microorganisms may have clinical consequences.

The duration of anti-infective therapy was not different between patients with and without bacteraemia. The optimal duration of antibiotic therapy for bacteraemia during intra-abdominal infections is unknown. In our study, antibiotic therapy for bacteraemic PIAI was administered for 11 ± 6 days, a duration usually reported in the literature. Our data suggest that extended therapy is not required in bacteraemic patients. However, this study was not tailored to address this issue and any definitive conclusions would be premature since, in a recent study analysing the benefit of short-course antibiotic therapy in PIAI, we reported an increased risk of postoperative bacteraemia and a higher risk of treatment failure in the case of short-course therapy [28].

Several limitations to the present study must be mentioned, including its retrospective nature. The results of this single-centre analysis may not be directly applicable to other institutions. Breakthrough infections and recurrent bacteraemia could not be assessed due to the short time window for analysis of bacteraemia. The long study duration is also a factor to be taken into account, with changing patient characteristics over recent years, with older and more severely ill patients, but the year of inclusion was adjusted for in the multivariate models. The quality of infection source control was not evaluated in this study, although it is an essential element of the prognosis of PIAI patients. The number of reoperations, the reasons for these operations, and the surgical findings did not differ between bacteraemic and non-bacteraemic patients, suggesting that bacteraemia did not play a major role in the outcome.

## Conclusion

In this single-centre observational study, one-fifth of patients had bacteraemia during the early management of PIAI. Bacteraemia was not a risk factor for mortality compared with patients without bacteraemia, and morbidity criteria did not appear to be modified. None of the mortality risk factors identified in our study are susceptible to corrective measures designed to improve the management of these critically ill patients. Increasing the duration of anti-infective therapy could have been proposed to improve the prognosis, although the current trend is to reduce the duration of therapy. Early diagnosis and early management of organ failures and their consequences remain the best rules in the treatment of these patients.

## Abbreviations

CI: Confidence interval
CNS: Coagulase-negative staphylococci
EAT: Empirical antibiotic therapy
ICU: Intensive care unit
IQR: Interquartile range
MDR: Multidrug-resistant
OR: Odds ratio
PIAI: Postoperative intra-abdominal infection
SAPS: Simplified Acute Physiology Score
SOFA: Sequential Organ Failure Assessment
